# Complete chloroplast genome of *Leptopus chinensis* (Bunge) Pojark (Euphorbiaceae), a traditional Chinese herbal medicine

**DOI:** 10.1080/23802359.2022.2132838

**Published:** 2022-10-20

**Authors:** Zhen-Ning Zhao, Xiao Yu

**Affiliations:** a School of Forestry, Southwest Forestry University, Kunming, China; b School of Landscape Architecture and Horticulture Sciences, Southwest Forestry University, Kunming, China

**Keywords:** *Leptopus chinensis* Pojark, chloroplast genome, phylogenetic analysis

## Abstract

*Leptopus chinensis* (Bunge) Pojark is a medicinal herb endemic to China. In this study, its complete chloroplast (cp) genome was characterized with a discussion of its phylogenetic placement. The cp genome of *L. chinensis* was 154,600 bp long, with a double-stranded circular tetrad structure containing a small single-copy (SSC) region (17,717 bp), a large single-copy (LSC) region (83,159 bp), and a pair of inverted repeat (IR) regions (26,862 bp each). The overall GC content of the genome was 36.8% (LSC, 34.7%; SSC, 29.8%; IR, 42.3%). Phylogenetic analysis indicated that *L. chinensis* is a sister species to *L. cordifolius*.

*Leptopus chinensis* (Bunge) Pojark (1833), a medicinal herb widely distributed in many regions of China, is commonly used to treat diseases such as jaundice, gastritis, and edema (Liao et al. 2003). Through *in vitro* experiments with aqueous and ethanol extracts of *L. chinensis* on Eca-109 cells, *L. chinensis* extract was demonstrated to exert a strong inhibitory effect on tumor cells (Lu et al. 2000; Yue et al. 2000; Long et al. 2002), indicating its high medicinal potential in the treatment of cancer. The chloroplast (cp) genome plays a vital role in determining species identification, phylogenetic relationships, and germplasm diversity. However, to date, the cp genome of *L. chinensis* has not been reported, and current studies have mainly concentrated on its medicinal value and chemical activity. Consequently, the complete cp genome of *L. chinensis* was characterized in this study, providing important information for phylogenetic, genetic diversity, and evolutionary studies of this valuable herb.

Fresh leaves of *L. chinensis* were collected from Yeri Village, Benzilan Town, Deqin County, Diqing Tibetan Autonomous Prefecture, Yunnan Province, China (99°8′23.07″E, 28°24′3.94″N; altitude: 2485 m). Plant samples were collected in accordance with the wild plant protection regulations of the People’s Republic of China and were approved by the Forestry and Grassland Bureau of Yunnan Province. A voucher specimen (SWFU20210780MFY) was deposited in the herbarium of Southwest Forestry University, China (http://bbg.swfu.edu.cn/, Xiao Yu, email: yuxiao0215@gmail.com). Total DNA was extracted from dried plant leaf specimens using a modified cetyltrimethylammonium bromide method and purified (Doyle and Doyle 1987). The purified DNA sample was then used to prepare the DNA sequencing library, and high-throughput paired-end sequencing (2 × 150 bp) was performed on an Illumina HiSeq 4000 platform (Illumina, San Diego, CA) by Tianjin Novogene Biotechnology Co., Ltd. (Tianjin, China). Totally, 3 Gbp of raw sequencing data was retrieved, and was used to assemble the cp genome using GetOrganelle (Jin et al. 2020) with the following parameters: wordize = 102; base coverage = 171.44; and *k* = 75, 85, 95, 105, 115, and 127. Annotation of the resultant cp genome was done using the online tool CPGAVAS2 (Shi et al. 2019), and was further manually improved in Geneious Prime (Kearse et al. 2012). The annotated cp genome of *L. chinensis* was submitted to GenBank under the accession number ON050995.

The complete cp genome of *L. chinensis* is 154,600 bp in total length, with a double-stranded circular tetrad structure comprising a small single-copy (SSC) region with a length of 17,717 bp, a large single-copy (LSC) region with a length of 83,159 bp, and a pair of inverted repeat (IR) regions with a length of 26,862 bp. The overall GC content of the genome was 36.8% (LSC, 34.7%; SSC, 29.8%; IR, 42.3%), and those of A, T, C, and G were 31.24%, 31.99%, 18.68%, and 18.09%, respectively. A total of 128 coding genes, including one pseudogene, 82 protein-coding genes (PCGs), eight ribosomal RNA genes (rRNAs), and 37 transfer RNA genes (tRNAs), were annotated. Using the online software MISA-web (Beier et al. 2017) with default parameters, 82 SSRs were detected, of which 57, 14, 4, 7, and 0 were mono-, di-, tri-, tetra-, and pentanucleotide SSRs, respectively.

A phylogenetic tree was constructed based on the cp genome of *L. chinensis* and 15 species of Euphorbiaceae, with *Taraxacum hallaisanense* (NC_057107) and *Lactuca sativa* (AP007232) as the outgroup (Figure 1). The MAFFT software (Katoh and Standley 2013) was used for multiple alignment among the cp genomes of these 18 species (scoring matrix = 200; PAM *k* = 2; gap open penalty = 1.53; offset value = 0.123). Subsequently, the resultant alignment was checked using MEGA v.11 software (Tamura 2021), and exported into the *.NET format. An maximum-likelihood (ML) phylogenetic tree was constructed using RAxML ver.8.0.0 (Stamatakis 2014) with the following parameters: bootstrap = 1000 and m = GTR + GAMMA. The phylogenetic tree was visualized using FigTree ver.1.4.3 software (http://tree.bio.ed.ac.uk/software/figtree/). The result revealed that all Euphorbiaceae species formed a monophyletic clade. In addition, *L. chinensis* and *L. cordifolius* were shown to be sister species. Our cp genome data of *L. chinensis* would contribute to future studies of taxonomy, genetics and genomics of related taxa.

**Figure 1.**
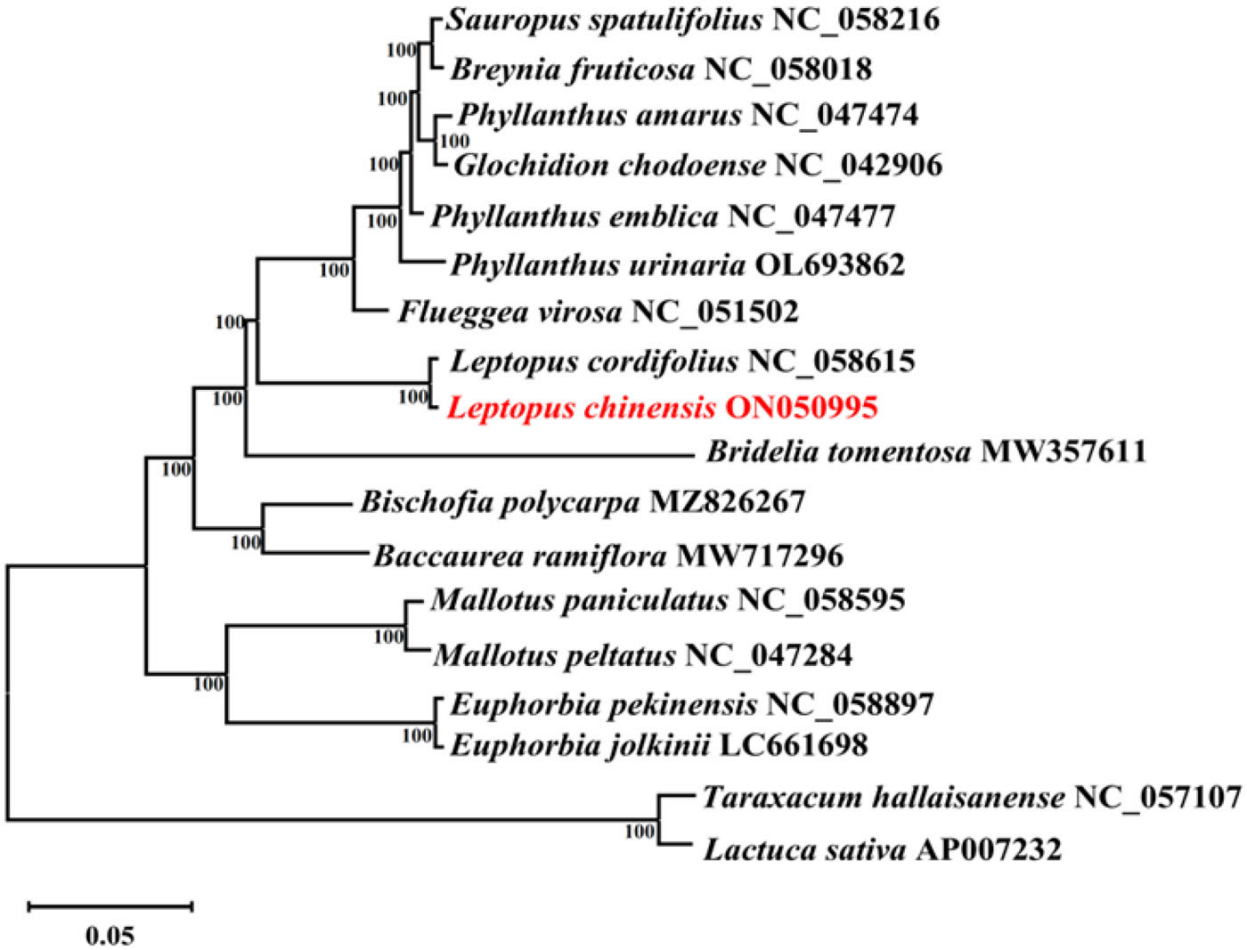
ML phylogenetic tree based on complete chloroplast genome of *L. chinensis* and 17 other species. Numbers in the nodes are the bootstrap values from 1000 replicates. The following sequences were used: *Sauropus spatulifolius* NC_058216, *Breynia fruticosa* NC_058018 (Zhou et al. 2020), *Phyllanthus amarus* NC_047474, *Glochidion chodoense* NC_042906, *Phyllanthus emblica* NC_047477, *Phyllanthus urinaria* OL693862, *Flueggea virosa* NC_051502, *Leptopus cordifolius* NC_058615 (Rehman et al. 2021), *Leptopus chinensis* ON050995, *Bridelia tomentosa* MW357611, *Bischofia polycarpa* MZ826267, *Baccaurea ramiflora* MW717296 (Niu and Liu 2022), *Mallotus paniculatus* NC_058595, *Mallotus peltatus* NC_047284, *Euphorbia pekinensis* NC_058897, *Euphorbia jolkinii* LC661698 (Iwata et al. 2022), *Taraxacum hallaisanense* NC_057107 (Lee et al. 2021), and *Lactuca sativa* AP007232.

## Data Availability

The data that are newly obtained at this study are available in the NCBI under accession number of ON050995 (https://www.ncbi.nlm.nih.gov/nuccore/ON050995). The associated BioProject, SRA, and Bio-Sample numbers are PRJNA820363, SRR18494790, and SAMN26994331, respectively.
